# Brown grease-derived ester base oils: tunable viscosity and cold-flow behaviour

**DOI:** 10.1039/d6ra02359b

**Published:** 2026-07-03

**Authors:** Ykok B. Ksor, Ashlyn D. Smith, Rhett C. Smith

**Affiliations:** a Department of Chemistry, Clemson University Clemson SC 29634 USA rhett@clemson.edu

## Abstract

Brown grease (BG) was converted into fatty acid methyl (FAMEc), ethyl (FAEEc), and isopentyl (FAPEc) esters, then epoxidized or hydroxylated to generate seven bio-based ester base oils: FAPEc, h-FAPEc, h-FAEEc, h-FAMEc, e-FAPEc, e-FAEEc, and e-FAMEc. Thermal stability (*T*_d,5%_), kinematic viscosity (*ν*_40_, *ν*_100_), viscosity index (VI), pour point, and crystallization behavior (DSC) were evaluated. Transesterification reduced viscosity (*ν*_40_ of 7.51–11.7 mm^2^ s^−1^) and improved cold-flow performance relative to crude BG, with FAPEc pour point of −6 ± 3 °C, consistent with suppressed crystallization observed by DSC. Subsequent hydroxylation and epoxidation enabled viscosity tuning across ISO VG 15-460 grades. Hydroxylated derivatives increased viscosity (*ν*_40_ of 12.6–30.7 mm^2^ s^−1^) with moderate pour point penalties (−3 to 8 ± 3 °C), whereas epoxidized derivatives produced substantial viscosity increases with ester cap-dependent cold flow performance. Notably, e-FAPEc achieved ISO VG 68 while maintaining a pour point of 3 ± 3 °C, demonstrating improved cold-flow performance compared with previously reported epoxy-BG systems. Tribology (ASTM D4172) testing showed ester-platform-dependent behavior, with h-FAEEc giving the lowest average friction, and FAPEc the poorest wear resistance. These results demonstrate that ester platform selection provides a practical design handle for tuning viscosity grade and low-temperature performance in brown grease-derived base oils.

## Abstract 2

Brown grease (BG) was converted into fatty acid methyl (FAMEc), ethyl (FAEEc), and isopentyl (FAPEc) esters, then epoxidized or hydroxylated to produce seven bio-based ester base oils: FAPEc, h-FAPEc, h-FAEEc, h-FAMEc, e-FAPEc, e-FAEEc, and e-FAMEc.

## Graphical abstract

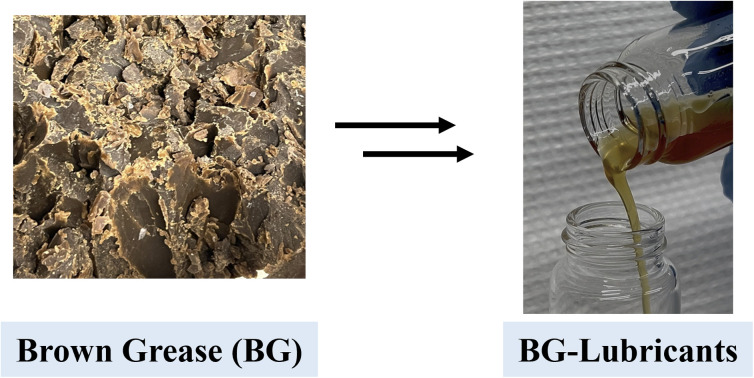

## Introduction

Industrial lubricants are essential to manufacturing, transportation, and energy systems by helping manage wear, heat stress, and surface protection.^2–7^ The majority of commercial base oils are derived from petroleum feedstocks, adding to environmental concerns and sustainability.^8,9^ The need for sustainable materials has motivated the investigation of bio-based alternatives capable of matching the viscosity, thermal stability, and low-temperature performance required for industrial applications.^10–16^ Bio-based oils, prepared from edible plant oils (*e.g.*, soybean, canola, palm), have been designed, providing a greener lubricant platform.^17–25^ However, the use of plant-based oils intensifies competition with food systems and land use, potentially increasing commodity prices and raising concerns around food insecurity.^26^ Therefore, it is important to find alternatives to edible feedstocks for non-edible goods.^27–29^ Triglyceride-derived oils offer high lubricity and favorable viscosity indices (VI) but often suffer from limited oxidative stability and poor cold-flow behavior due to efficient chain packing and crystallization.

Brown grease (BG) is a low-value waste stream generated from food service operations, industrial meat processing, and wastewater treatment facilities. Brown grease has a high free fatty acid (FFA) content and may contain tri-, di-, and monoglycerides, water, solids, phospholipids, pigments, and trace metals, making it unsuitable for use in nutritional products for human or animal markets.^30–32^ Brown grease is produced as a waste product and does not require agricultural land. A 2020 National Renewable Energy Laboratory study estimated that brown grease costs $100 USD per dry metric ton, compared to ∼$1000 USD per ton of soybean oil, making it an abundant, cost-efficient, and underutilized lipid feedstock,^33^ despite recent efforts to utilize waste lipid feedstocks for polymers,^34,35^ lubricants^36–38^ or structural composites.^1,39–43^

**Scheme 1:**
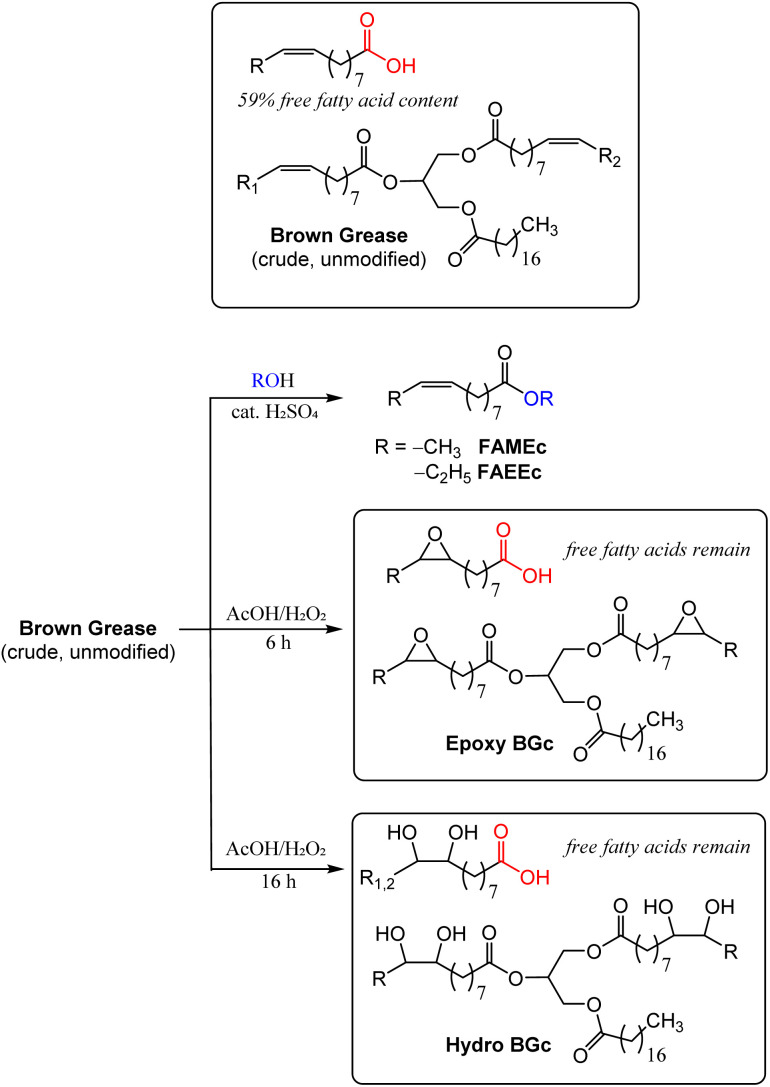
Chemical structures of BG-derived base oils.

In previous work on BG-derived lubricants (Scheme 1), one-pot functionalization *via* esterification, epoxidation, and hydroxylation of crude and bleached brown grease produced brown grease-derived base oils spanning ISO VG 10 to >1500, demonstrating significant viscosity amplification.^1^ Results from the previous study showed that esterification to give FAEEc and FAMEc leads to reduced viscosity and improved cold-flow performance (*ν*_40_ of 12 mm^2^ s^−1^, pour point = 3 °C). Oxidative derivatization to create Epoxy BGc and Hydro BGc resulted in significant increases in viscosity at the cost of unsuitable cold-flow performance (*ν*_40_ of 98–1700 mm^2^ s^−1^, pour point = 28–41 °C). These findings suggest that the presence of FFAs significantly affects crystallization behavior, leading to higher cold-crystallization temperatures and poor cold-flow performance.

**Scheme 2:**
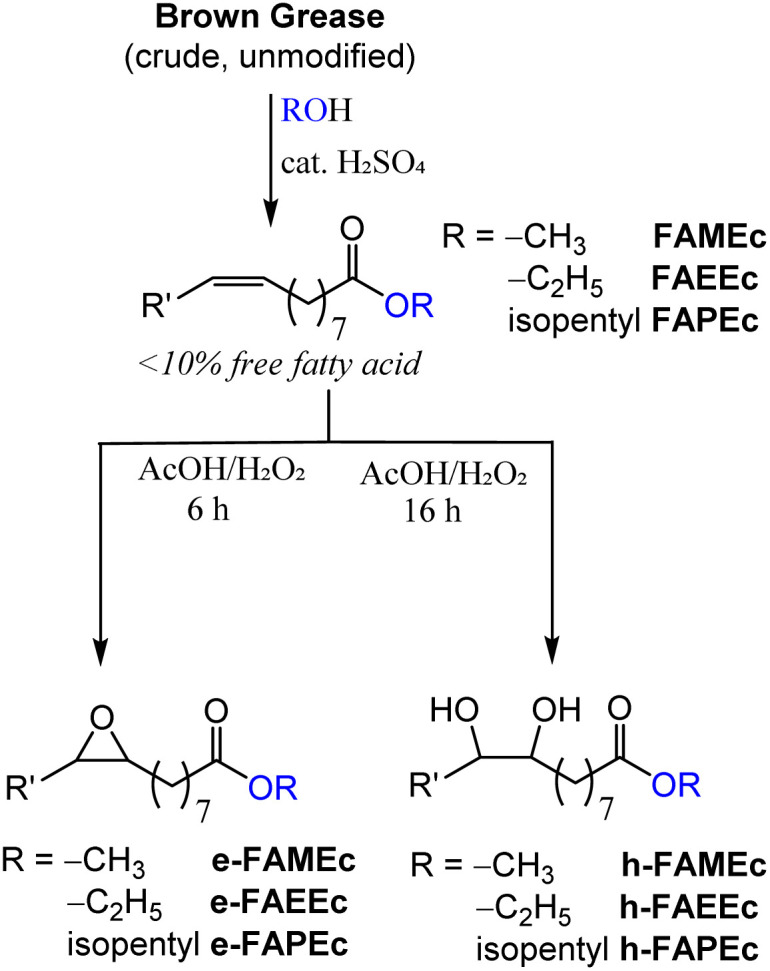
Chemical modification of BG alkyl esters by esterification, epoxidation, and hydroxylation.

Previous findings have shown improved cold-flow performance through esterification of carboxylate derivatives.^1,44^ The present study thus seeks to address cold-flow performance issues by introducing a platform-based strategy in which BG is first fully esterified to remove free fatty acids and disrupt the triglyceride framework, generating defined fatty acid methyl (FAMEc), ethyl (FAEEc), and isopentyl (FAPEc) esters (Scheme 2). Isoamyl alcohol was chosen to expand the set of brown grease-derived esters based on prior work suggesting that low-temperature performance could be improved with a branched alkyl ester substituent.^45^ Subsequent hydroxylation or epoxidation of each of the three esters was also undertaken to assess the effect on viscosity. This approach was designed to provide a tunable ISO viscosity grade range, quantify low-temperature behavior through differential scanning calorimetry (DSC) and pour point analysis, and establish structure–property relationships linking ester cap identity and functional group incorporation to rheological performance.

Herein, we report thermal stability (*T*_d,5%_), dynamic and kinematic viscosities (*µ*_40_, *µ*_100_; *ν*_40_, *ν*_100_), viscosity index (VI), crystallization and melting transitions (DSC), and pour point for the parent esters and their oxidative derivatives. The results are benchmarked against previously reported BG-derived materials and evaluated within commercial ISO VG target windows. Through direct comparison of “same functional group, different alcohol” and “same alcohol, different functional group,” the influence of ester platform selection on viscosity amplification and cold-flow behavior is established. This work provides a framework for developing waste-derived base oils from BG with tunable viscosities and improved cold-flow performance.

## Results and discussion

### Synthesis and initial characterization

Brown grease was subjected to fatty acid profile testing, revealing a fatty acid composition of 44.85 wt% oleic acid, 21.67 wt% palmitic acid, 17.65 wt% linoleic acid, and 6.29 wt% stearic acid, and <5% of other fatty acid chains. Overall composition showed 50.54 wt% monounsaturated, 29.43 wt% saturated, and 20.03 wt% polyunsaturated content. The brown grease also had free fatty acid content of 59%, a peroxide value of 10 mmol O_2_ per kg oil, and an iodine value of 280 g I_2_/100 g oil as we previously reported.^1^ A detailed composition of the brown grease is shown in SI (SI Table S1).

Material abbreviations used herein are defined. BG refers to brown grease, with subscripts “c” and “b” denoting crude and bleached feedstock. FAMEc, FAEEc, and FAPEc designate fatty acid methyl, ethyl, and isopentyl esters derived from BGc. The prefixes “e-” and “h-” denote epoxidized and hydroxylated derivatives for e-FAMEc, e-FAEEc, e-FAPEc, h-FAMEc, h-FAEEc, and h-FAPEc, respectively. The synthetic routes to all newly prepared esterified oils and their oxidative derivatives evaluated in this study are summarized in Scheme 2. Previously reported base oils made from BG, such as Hydro BGc, Hydro BGb, Epoxy BGc, and Epoxy BGb are reported consistently with previous labels.^1^

**Fig. 1:**
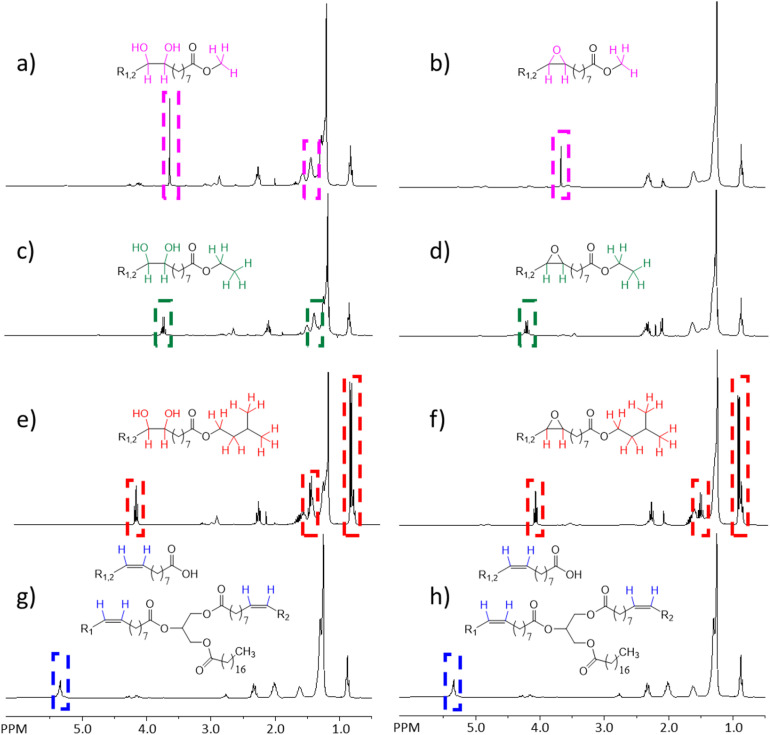
Proton NMR spectra (300 MHz, CDCl_3_) of (a) h-FAMEc, (b) e-FAMEc, (c) h-FAEEc, (d) e-FAEEc, (e) h-FAPEc, (f) e-FAPEc, and (g and h) BGc. Dashed boxes represent the proton signals corresponding to the olefins (blue), and the methine, methylene, and methyl units from the ester substituent and respective hydroxylation or epoxidation (red, green, pink).

Successful conversion of BG-derived ester base oils to their hydroxylated and epoxidized derivatives was confirmed by ^1^H NMR spectroscopy (300 MHz, CDCl_3_) as seen in Fig. 1 (SI 1–7). In all cases, the disappearance of the olefinic resonances centered at 5.34 ppm indicated full consumption of olefins present in the fatty acid chains. Concomitantly, new resonances corresponding to oxidative functionalization were observed. For hydroxylated and epoxidized samples, characteristic methine and methylene signals emerged around 1.50 ppm, with the hydroxylated samples displaying a more defined peak. Stability of the ester when undergoing oxidative functionalization was confirmed by the maintenance of methylene and methyl resonances attributable to the ester substituents.

### Thermal properties (TGA, DSC, pour point)

Thermogravimetric analysis (TGA) shows decomposition behaviours of respective base oils as seen in Table 1 and SI 8. The parent esters exhibit *T*_d,5%,_ ranging from 183 to 205 °C. Hydroxylated derivatives showed slightly increased thermal stability, with *T*_d,5%_ ranging from 194 to 210 °C. The epoxidized counterparts displayed similar thermal stability to that of parent esters, with *T*_d,5%_ ranging from 187 to 206 °C. Across all the newly designed base oils, no significant changes in thermal stability are observed. However, FAEEc- and FAMEc-based materials, including their oxidative derivatives, exhibit a significant second mass-loss event near 325 °C. In contrast, FAPEc-based materials show a single dominant decomposition event near their *T*_d,5%_ values primarily. These results indicate that the ester substituent can influence the degradation profile of the designed base oils.

**Fig. 2:**
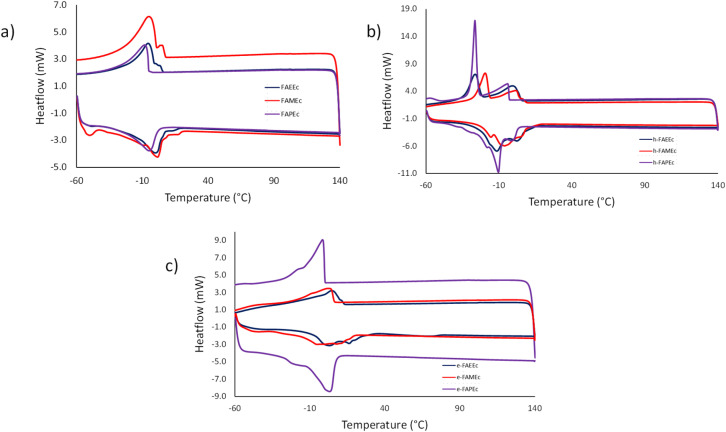
Differential scanning calorimetry traces for (a) FAEEc, FAMEc, FAPEc, (b) h-FAEEc, h-FAMEc, h-FAPEc, and (c) e-FAEEc, e-FAMEc, e-FAPEc. Third heating and cooling cycles are shown with endothermic pointed down in these thermograms.

Thermograms obtained by differential scanning calorimetry (DSC) for parent esters and their oxidative derivatives are shown in Fig. 2. Parent esters exhibit crystallization onsets near −3 °C, with crystallization peak temperatures observed at −4 °C for FAEEc and FAMEc, and −5 °C for FAPEc. Melting onsets for parent esters are observed near 6 °C for FAEEc and FAMEc, and −3 °C for FAPEc, with melting peak temperatures reported at −5 °C for FAEEc and FAMEc, and −9 °C for FAPEc, respectively. Differences in crystallization and melting peak shapes are also observed among the parent esters. FAEEc and FAMEc display a secondary shoulder overlapping with primary crystallization and melting peaks, suggesting the presence of multiple ordering populations that can be attributable to the mixed fatty acid ester distribution. This behaviour is consistent with compositional heterogeneity arising from variations in chain length and saturation. In contrast, FAPEc exhibits a single, well-defined crystallization and melting peak, indicating that the isopentyl ester substituent disrupts the packing environment more than the methyl and ethyl analogues, perhaps due to its bulkier nature.

Changes in the crystallization and melting behaviours of the hydroxylated parent esters are observed. Crystallization onsets for h-FAEEc and h-FAMEc are at 17 °C, with h-FAPEc reported at 3 °C. The higher crystallization onset is consistent with increased intermolecular interactions from the addition of the OH groups. Crystallization peak temperatures for h-FAEEc are −9 and 0 °C, −8 °C for h-FAMEc, and −11 and −1 °C for h-FAPEc. Melting onsets for hydroxylated parent esters are 3 °C for h-FAEEc and h-FAMEc, and −3 °C for h-FAPEc. The hydroxylated parent esters also had two distinct melting peaks: h-FAEEc at 0 and –26 °C, h-FAMEc at 3 and –19 °C, and h-FAPEc at −4 and −26 °C. The presence of two crystallization and melting peaks is in line with expectations and reasoning previously mentioned for the parent esters. The change of h-FAPEc to show two distinct peaks for crystallization and melting suggests the influences of the added OH groups to counterbalance the disrupting packing nature of the bulky isopentyl ester substituent.

Epoxidized parent esters also show changes in crystallization and melting behaviours. Significant broadening of crystallization peaks is observed for both e-FAEEc and e-FAMEc, indicating compositional dispersion amplified by epoxidation. However, e-FAPEc, maintains its peak shape, highlighting once again the effects of the isopentyl ester substituent as described previously. Crystallization onsets for e-FAEEc and e-FAMEc were reported at 32 and 19 °C, respectively, with e-FAPEc reported at 8 °C. Crystallization peak temperatures for e-FAEEc are 16 and 4 °C, 2 °C for e-FAMEc, and 3 °C for e-FAPEc. Changes in crystallization peak temperature for FAPEc and its oxidative derivatives are minimal compared with the observed shifts in FAEEc and FAMEc, and their oxidative derivatives.

Pour point data can provide information on the cold-flow behaviour of our BG-derived base oils, with observed pour points ranging from 3–41 °C. Comparison of pour point data with DSC analysis shows correlative crystallization behaviour. Parent esters exhibit pour points of 3 ± 3 °C for FAEEc and FAMEc, and −6 ± 3 °C for FAPEc, consistent with their crystallization onsets near −3 °C. Hydroxylated derivatives showed increased pour points of 8 ± 3 °C for h-FAEEc and h-FAMEc, and −3 ± 3 °C for h-FAPEc, corresponding to elevated crystallization onset temperatures observed in the DSC traces. The epoxidized derivatives showed increased pour points as well, with 41 ± 3 °C for e-FAEEc, 13 ± 3 °C for e-FAMEc, and 3 ± 3 °C for e-FAPEc. The lower observed pour points of the isopentyl ester parent oil and its derivatives in comparison to the other ester substituents indicate that the identity of the ester can have a significant influence on low-temperature flow even after oxidative modifications.

### Rheology and viscosity grade tuning

Kinematic (*ν*) viscosities at 40 and 100 °C, viscosity index (VI), and ISO VG classifications for the BG-derived base oils are summarized in Table 1 and 2. Comparison of designed base oils by ester substituent and oxidative functionalization reveals structure–property relationships affecting viscosity and low-temperature flow.

**Table 1:** Thermal and rheological properties of brown grease and derived oils. Thermal properties reported as temperature at 5% weight loss (*T*_d,5%_).^a^

Sample name	*T*_d,5%_ (°C)	Char yield at 800 °C	Dynamic viscosity (mPa s)		Density (g mL^−1^)		Kinematic viscosity (mm^2^ s^−1^)		Viscosity index (VI)	Pour point (°C)
		(wt%)	40 °C (*µ*_40_)	100 °C (*µ*_100_)	40 °C (*ρ*_40_)	100 °C (*ρ*_100_)	40 °C (*ν*_40_)	100 °C (*ν*_100_)		
Brown grease (crude)	209	0	35	6.68	0.92	0.89	38.0	7.51	170	28
Brown grease (bleached)	209	0	31.3	5.05	0.92	0.87	34.0	5.80	112	31
FAEEc	200	0	10.4	2.52	0.888	0.868	11.7	2.90	93.6	3
FAEEb	196	0	8.35	2.52	0.895	0.845	9.33	2.98	199	0
FAMEc	190	0	10.5	2.52	0.898	0.861	11.7	2.93	99.8	3
FAMEb	183	0	8.45	2.15	0.881	0.854	9.59	2.52	83.1	3
FAPEc	205	5	6.55	1.70	0.872	0.836	7.51	2.03	41.2	−6
Hydro BGc	234	0	95.4	11.5	0.97	0.92	98.4	12.5	121	28
Hydro BGb	215	0	95.5	7.55	0.962	0.921	99.3	8.20	12.4	28
h-FAMEc	206	3	22.6	3.94	0.950	0.903	23.8	4.36	83.3	8
h-FAEEc	194	1	28.9	5.63	0.940	0.903	30.7	6.23	158	8
h-FAPEc	210	6	11.3	2.24	0.900	0.857	12.6	2.61	−12.1	−3
Epoxy BGc	211	0	1590	40.5	0.92	0.903	1730	44.9	30.3	41
Epoxy BGb	229	0	1440	39.3	0.946	0.901	1520	43.6	39.6	41
e-FAMEc	187	3	427	28.5	0.935	0.908	457	31.4	99.2	13
e-FAEEc	198	5	319	14.0	0.965	0.937	331	14.9	−26.2	41
e-FAPEc	206	2	60.8	6.87	0.933	0.896	65.2	7.67	74.6	3

a Lowercase “c” and “b” in sample names refer to crude and bleached brown grease, respectively.

**Table 2:** ISO viscosity-grade assignments and application notes for brown-grease-derived oils

Sample	ISO VG (by *ν*_40_)	Notes
Brown grease (crude)	32	High VI for a raw feed, but very high pour point; unsuitable neat
Brown grease (bleached)	32	Lower *ν* and VI *vs.* crude; poor cold flow
FAEEc	10	Light ester; VI modest, cold flow reasonable
FAEEb	10	Top candidate: very high VI (≈synthetic esters); cold flow ≈0 °C; light viscosity
FAMEc	10	Biodiesel-like methyl esters; light & modest VI
FAMEb	10	Lower VI and *ν*; light stock with limited temperature robustness
FAPEc	10 (below VG 10 range)	Very low viscosity; poor VI but improved cold flow ≈−6 °C
Hydro BGc	100	ISO VG 100 with decent VI & high *T*_d_
Hydro BGb	100	VI collapses after bleaching; unusable neat
h-FAMEc	22	Modest VI (≈group I); poor cold flow ≈8 °C; light viscosity
h-FAEEc	32	High VI (≈synthetic esters); poor cold flow ≈8 °C; light viscosity
h-FAPEc	15	Extremely poor VI; slightly improved cold flow ≈−3 °C
Epoxy BGc	>1500 (classify as thickener)	Extremely viscous, poor VI; use as viscosity modifier only
Epoxy BGb	>1500 (classify as thickener)	Similar to crude epoxy; too viscous for base-oil use
e-FAMEc	460	Viscous; moderate VI; poor cold flow ≈13 °C
e-FAEEc	320	Extremely viscous, very poor VI, unsuitable neat
e-FAPEc	68	Moderate viscosity, VI modest, adequate cold flow

Parent esters show that the introduction of the ester cap lowers viscosity and improves cold-flow behaviour. FAEEc and FAMEc exhibit a *ν*_40_ of 11.7 mm^2^ s^−1^ (ISO VG 10 classification) and pour point at 3 ± 3 °C. This highlights a significant reduction in viscosity and improved cold-flow behaviour compared to starting BGc feedstock with a *ν*_40_ of 38.0 mm^2^ s^−1^ (ISO VG 32 classification) and pour point of 28 ± 3 °C. FAPEc follows the trend more significantly with a *ν*_40_ of 7.51 mm^2^ s^−1^ (below VG 10 range) and an improved pour point of −6 ± 3 °C relative to the other ester substituents. This is consistent with the disrupted packing environment observed and discussed in the DSC analysis and indicates that incorporation of the bulkier isopentyl ester substituent suppresses crystallization and improves cold-flow performance at the expense of reduced viscosity. An ISO VG rating of 10 for the parent esters indicates a very light oil resembling spindle-oil-class fluids^46–48^ and other synthetic ester base oils.^49^

Analysis of hydroxylated parent esters shows that the introduction of OH groups causes increases in viscosity and pour point. h-FAEEc and h-FAMEc exhibit a *ν*_40_ of 30.7 mm^2^ s^−1^ (ISO VG 32) and 23.8 mm^2^ s^−1^ (ISO VG 22), respectively, with a pour point of 8 ± 3 °C. This demonstrates a trend of esterification reducing viscosity and pour point by limiting the presence of FFA, which can contribute significantly to intermolecular forces that promote efficient packing, and the introduction of OH groups through hydroxylation that introduces more sites for intermolecular interactions, resulting in changes in crystallization onset as mentioned previously. This leads to the observed trend of the parent ester with the lowest viscosity and pour point, the hydroxylated parent esters with an intermediate viscosity and pour point in comparison to their parent ester and starting crude brown grease feedstock (BGc), with the highest viscosity and pour point. This trend can also be seen with h-FAPEc, reporting a *ν*_40_ of 12.6 mm^2^ s^−1^ (ISO VG 15), and a pour point of −6 ± 3 °C.

Epoxidation of parent esters produce a more pronounced viscosity increase. e-FAEEc and e-FAMEc have a *ν*_40_ of 331 mm^2^ s^−1^ (ISO VG 320) and 457 mm^2^ s^−1^ (ISO VG 460), respectively, with a pour point of 41 ± 3 °C and 13 ± 3 °C, respectively. The epoxidized parent ester shows a significant improvement in applicable viscosity in comparison to the Epoxy BGc reported previously,^1^ with a *ν*_40_ of 1730 mm^2^ s^−1^ (ISO VG > 1500), creating an unsuitable base oil that would initially be classified as a thickener, into a very heavy oil. This, however, comes at the cost of pour point as discussed previously, leading to unsuitability for lubricant applications. This is seen to be overcome by the identity of the parent ester, with e-FAPEc reporting a *ν*_40_ of 65.2 mm^2^ s^−1^ (ISO VG 68), and a pour point of 3 ± 3 °C. These results show that the cold-flow penalty associated with epoxidation is strongly ester cap-dependent.

Among the newly designed BG-derived base oils, e-FAPEc (ISO VG 68, VI 74.6, pour point 3 ± 3 °C) provides a balanced combination of improved viscosity and cold-flow performance in comparison to the other epoxidized derivatives while also having moderate VI. h-FAEEc (ISO VG 32, VI 158, pour point 8 ± 3 °C) offers a high VI synthetic ester with modest pour point in comparison to other hydroxylated brown grease derivatives. In contrast, e-FAEEc, and e-FAMEc, while having high viscosities, exhibit elevated pour points that limit neat base oil applicability without additive modifications.

### Benchmarking *vs.* prior BG-derived materials and commercial “target windows”

The newly designed BG-derived base oils have ISO VG ratings ranging from 10 to 460. Across all BG-derived base oils highlighted in Table 2, the range expands to greater than 1500. The vast ISO VG range highlights the versatility of brown grease modification through transesterification and subsequent oxidative derivatization. Transesterification provides a platform for various ester derivatives that can be utilized to improve cold-flow performance, as seen with FAPEc, which has the best reported pour point among all BG-derived base oils in Table 2. On its own, FAPEc is a very light oil, but subsequent oxidative modifications can increase the viscosity of the material with minimal increases in cold-flow performance, as seen with e-FAPEc. An ISO VG rating of 68 for e-FAPEc puts it in the range of other synthetic base oils and other commercial oils used in hydraulic systems.^50,51^

This work establishes that parent esters derived from brown grease, combined with oxidative functionalization, enables tunable viscosity and cold-flow performance to create new sustainable base oils with comparable ISO VG ratings to other commercial base oils. The removal of FFA through esterification to improve cold-flow performance and the oxidative functionalization to improve viscosity provides a simple and efficient pathway to generate sustainable base oils. However, several factors remain to be evaluated for full lubricant applicability, such as wear performance through tribology, and oxidative stability. The work shown above also highlights the limitations of cold-flow performance for BG-derived base oils in comparison to other synthetic ester base oils with pour points as low as −60 °C.^52,53^ Further avenues of research should explore improving the cold-flow performance of brown grease-derived base oils by introducing additives, such as pour point depressants (PPD).

### Tribology

Preliminary tribological tests were performed using a four-ball wear configuration (ASTM D4172; 40 kg, 1200 rpm, 75 °C, 2 h, coefficient of friction *versus* time plots provided in SI Fig. S9–11) as in prior work on brown grease-derived lubricants^1^ for three representative ester-platform candidates, h-FAEEc, FAPEc, and e-FAPEc. The measured wear scar diameters were 0.90 mm for h-FAEEc, 1.02 mm for FAPEc, and 0.80 mm for e-FAPEc, with corresponding grand-average coefficients of friction of 0.059, 0.082, and 0.092, respectively. The h-FAEEc trace showed an initial transient followed by stabilization near 0.05–0.06, whereas FAPEc decreased rapidly before levelling off near 0.08 and e-FAPEc maintained the highest friction over most of the test despite affording the smallest wear scar. Compared with the previously reported FAEEc and Hydro BGc benchmarks,^1^ which gave wear scar diameters of 0.80 and 0.70 mm and stabilized coefficients of friction of approximately 0.075 and 0.05, respectively, the present materials reveal a tunable friction-wear trade-off rather than a universal improvement. Hydroxylation of the ethyl ester lowers friction relative to FAEEc and approaches the low-friction behaviour of Hydro BGc, albeit with a larger wear scar, while epoxidation of the isopentyl ester substantially improves wear relative to FAPEc (1.02 to 0.80 mm) but increases average friction. FAPEc, despite its best cold-flow behaviour in the present series, showed the poorest wear resistance. Notably, e-FAPEc matches the wear scar of previously reported FAEEc while offering a much higher viscosity grade (ISO VG 68 *versus* ISO VG 10) and maintaining a pour point of 3 ± 3 °C, underscoring the utility of ester-cap selection for balancing viscosity, low-temperature performance, wear, and friction in BG-derived base oils.

## Conclusions

Brown grease (BG) was modified in two steps: transesterification, followed by epoxidation or hydroxylation to give seven new BG-derived base oils, which were evaluated for *µ*_40_/*µ*_100_, *ν*_40_/*ν*_100_, *ρ*, VI, pour point, and *T*_d,5%_. Tunability of viscosity and cold-flow performance was observed.

Transesterification of BGc to form FAEEc, FAMEc, and FAPEc reduced viscosity and improved pour point relative to crude feedstock, highlighting the removal of free fatty acids as an effective strategy to enhance cold-flow performance. More specifically, FAPEc showed that incorporation of a bulkier isopentyl substituent resulted in the lowest reported pour point (−6 °C ± 3 °C) amongst the BG-derived base oils.

Subsequent hydroxylation and epoxidation improve viscosity across the modified parent esters. The parent esters group around ISO VG 10 (*ν*_40_ = 7.51–11.7 mm^2^ s^−1^), with subsequent oxidative derivatizations shifting the hydroxylated and epoxidized materials to ISO VG 15–32 (*ν*_40_ = 21.6–30.7 mm^2^ s^−1^) and ISO VG 68-460 (*ν*_40_ = 65.2–457 mm^2^ s^−1^), respectively. The two-step chemical modification allows for fine-tuning of ISO VG grade, enabling access to a range of various lubricants. High-VI light esters, such as FAEEc and FAMEc, can be used for high-speed, low-load-bearing applications, typical of spindle-oil-class fluids.^46^ Mid-viscosity esters, such as h-FAEEc and e-FAPEc, can be used for various hydraulic systems.^50^ Preliminary four-ball tribology further showed that oxidative functionalization tunes the friction – wear balance, with h-FAEEc approaching the low-friction behaviour of Hydro BGc and e-FAPEc restoring wear resistance to the level of previously reported FAEEc while retaining ISO VG 68 viscosity.

Overall, the work described in this study provides an efficient two-step chemical modification of BG to create a platform of various esterified oils, highlighting tunability for application and a resourceful, cheap, bio-based feedstock for the design of base oils. Future work will explore long-term oxidative and shear stability, as well as the incorporation of additives, such as PPD, to improve cold-flow performance for the more promising lubricant candidates.

## Experimental

### Materials and methods

Reagents were used as received from Millipore Sigma, Acros Organics, and Thermo Fisher Scientific. Suppliers for brown grease were omitted to avoid endorsement or bias. Proton NMR spectra were obtained on a Bruker NEO-300 operating at 300 MHz at 20 °C–23 °C. Chemical shifts (*δ*) are reported in parts per million (ppm). Spectra data were processed with SpinWorks 4.2.11 software and referenced to residual solvent peak (^1^H, CDCl_3_, 7.26 ppm). Multiplicity is reported as s, singlet; d, doublet; t, triplet; q, quartet; and m, multiplet. Thermogravimetric analysis (TGA) was performed, and data were collected on a Mettler Toledo TGA 2 STARe System over the range of 25–800 °C, with a heating rate of 10 °C min^−1^ under a flow of N_2_ at a rate of 20 mL min^−1^. Dynamic viscosity (*µ*) at 40 and 100 °C was measured on an Anton Paar MCR 302e rheometer using measuring cone CP50–0.5; angle 0.5° geometry at a constant shear rate. Kinematic viscosity was calculated as *ν* = *µ*/*ρ*, and density (*ρ*) was calculated by volume displacement at 40 and 100 °C. Viscosity index (VI) calculated from *n*_40_ and *n*_100_ according to ASTM D2270. Pour point was measured per ASTM D97.^54^ Differential scanning calorimetry (DSC) data were acquired (Mettler Toledo DSC 3 STARe System) over a temperature range from −60 to 140 °C with a heating rate of 10 °C min^−1^ under a flow of N_2_ (200 mL min^−1^). Each DSC measurement was carried out over three heat-cool cycles.

#### Esterification to FAPEc

A 100 mL round-bottom flask equipped with a magnetic stir bar was placed in an oil bath on top of a magnetic stir plate. To the round-bottom flask, crude brown grease (20 g), isoamyl alcohol (200 mmol), and sulfuric acid (2 wt%) were added sequentially. The reaction flask was equipped with a condenser and heated to reflux at 70 °C for 5 hours. Upon completion of the reaction, the mixture was dissolved in 200 mL of diethyl ether, washed twice with 200 mL aqueous solution of 5 wt% sodium bicarbonate, followed by two washes with 200 mL of DI water. The organic phase was then collected, dried under magnesium sulfate for 15 minutes, vacuum-filtered to remove salts, and concentrated under reduced pressure to afford a dark brown oil.

#### Characterization of FAPEc

87% yield. Proton NMR [300 MHz, CDCl_3_, *δ* (ppm)]: 5.34 (m, **H**–C=C–, 2H); 4.09 (t, O–C**H**_**2**_, 2H); 2.28 (m, –C**H**_**2**_–CO, 2H); 2.01 (m, C**H**_**2**_–C=C–C**H**_**2**_, 3H); 1.69 (m, –CH_2_–C**H**–(CH_3_)_2_, 1H); 1.61 (m, –C**H**_**2**_–CCOO, 2H); 1.51 (q, –CH_2_–C**H**_2_–CH(CH_3_)_2_, 2H); 1.25 (m, –**CH**_**2**_–, 22H); 0.92 (d, CH–(C**H**_3_)_2_, 6H); 0.85 (m, –**CH**_**3**_, 3H).

#### General hydroxylation to h-FAEEc, h-FAMEc, and h-FAPEc

A 100 mL round-bottom flask equipped with a magnetic stir bar was placed in an oil bath on top of a magnetic stir plate. To the flask, 5 g of parent ester (FAEEc, FAMEc, or FAPEc) was added, heated to 60 °C, and agitated at 300 rpm. In a 20 mL scintillation vial containing a magnetic stir bar, formic acid (0.2 mL) was added and cooled in an ice bath with agitation at 100 rpm. To the cooled formic acid, 30 (w/w%) hydrogen peroxide (5 mL) was added dropwise over the course of 5 minutes. The solution was allowed to stir in the ice bath for an additional 5 minutes before being removed and stirred at room temperature for 15 minutes to allow the *in situ* formation of performic acid. The resulting performic acid solution was then added dropwise (1 drop every 3 seconds) to the pre-heated parent ester with continuous agitation at 60 °C and 300 rpm. Upon full addition, the reaction mixture was allowed to stir at 60 °C for 16 hours under ambient atmosphere. After completion, the reaction was allowed to cool to room temperature. To the flask, 25 mL of an aqueous solution of 5 wt% sodium bicarbonate and 5 wt% sodium sulfite was added slowly and agitated to neutralize residual acids and quench excess peroxide. The hydroxylated product was extracted twice with 50 mL portions of ethyl acetate. The combined organic extracts were dried over anhydrous magnesium sulfate for 15 minutes, vacuum-filtered to remove residual salts, and concentrated under reduced pressure to yield a viscous brown oil.

#### Characterization of h-FAEEc

96% yield. Proton NMR [300 MHz, CDCl_3_, *δ* (ppm)]: 4.13 (s, O–C**H**_2_CH_3_, 1H); 2.28 (m, –C**H**_**2**_–CO, 2H); 1.61 (m, –C**H**_2_–CCOO, 2H); 1.48 (m, –OCHC**H**_2_, 4H); 1.25 (m, –C**H**_2_–, 19H); 0.85 (m, –C**H**_3_, 3H).

#### Characterization of h-FAMEc

99% yield. Proton NMR [300 MHz, CDCl_3_, *δ* (ppm)]: 3.66 (s, O–C**H**_3_, 2H); 2.28 (m, –C**H**_**2**_–CO, 2H); 1.61 (m, –C**H**_2_–CCOO, 2H); 1.48 (m, –OCHC**H**_2_, 4H); 1.25 (m, –C**H**_2_–, 18H); 0.85 (m, –C**H**_3_, 3H).

#### Characterization of h-FAPEc

89% yield. Proton NMR [300 MHz, CDCl_3_, *δ* (ppm)]: 4.09 (t, O–C**H**_**2**_, 2H); 2.28 (m, –C**H**_**2**_–CO, 2H); 1.69 (m, –CH_2_–C**H**–(CH_3_)_2_, 1H); 1.61 (m, –C**H**_**2**_–CCOO, 2H); 1.51 (q, –CH–C**H**_2_–CH(CH_3_)_2_, not determined); 1.48 (m, –OCHC**H**_2_, not determined) (m 1.25 (m, –C**H**_**2**_–, 22H); 0.92 (d, CH–(C**H**_3_)_2_, 6H); 0.85 (m, –**CH**_**3**_, 3H).

#### General epoxidation to e-FAEEc, e-FAMEc, and e-FAPEc

A 50 mL round-bottom flask with a magnetic stir bar was placed in an oil bath on top of a magnetic stir plate. To the round-bottom flask, 5 g of parent ester (FAEEc, FAMEc, or FAPEc) was added, heated to 60 °C, and agitated at 300 rpm. In a 20 mL scintillation vial with a magnetic stir bar placed in an ice bath on top of a magnetic stir plate, glacial acetic acid (7 mL) and >98% sulfuric acid (0.2 mL) were added and agitated at 100 rpm. To the cooled acetic acid, 30 (w/w%) hydrogen peroxide (5 mL) was added dropwise. The solution in the vial was agitated in the ice bath for 20 minutes to allow the formation of the peracetic acid. The peracetic acid solution was then added dropwise (1 drop every 3 seconds) to the brown grease. Upon full addition of the peracetic acid solution, the resulting reaction mixture was allowed to stir at 300 rpm at 60 °C for 6 hours. Upon completion, the reaction was allowed to acclimate to room temperature. To the reaction flask, 50 mL of an aqueous solution of 5 wt% sodium bicarbonate and 5 wt% sodium sulfite was added slowly and agitated. The epoxidized product was then extracted twice with 50 mL of ethyl acetate. The organic phase was then collected, dried under magnesium sulfate for 15 minutes, vacuum-filtered to remove salts, and concentrated under reduced pressure to afford a brown oil that solidified into a wax upon cooling down to room temperature.

#### Characterization of e-FAEEc

94% yield. Proton NMR [300 MHz, CDCl_3_, *δ* (ppm)]: 4.13 (s, O–C**H**_2_CH_3_, 2H); 2.28 (m, –C**H**_**2**_–CO, 2H); 1.61 (m, –C**H**_2_–CCOO, 2H); 1.48 (m, –OCHC**H**_2_, not determined); 1.25 (m, –C**H**_2_–, 21H); 0.85 (m, –C**H**_3_, 3H).

#### Characterization of e-FAMEc

90% yield. Proton NMR [300 MHz, CDCl_3_, *δ* (ppm)]: 3.66 (s, O–C**H**_3_, 2H); 2.28 (m, –C**H**_**2**_–CO, 2H); 1.61 (m, –C**H**_2_–CCOO, 2H); 1.48 (m, –OCHC**H**_2_, not determined); 1.25 (m, –C**H**_2_–, 19H); 0.85 (m, –C**H**_3_, 3H).

#### Characterization of e-FAPEc

72% yield. Proton NMR [300 MHz, CDCl_3_, *δ* (ppm)]: 4.09 (t, O–C**H**_**2**_, 2H); 2.28 (m, –C**H**_**2**_–CO, 2H); 1.69 (m, –CH_2_–C**H**–(CH_3_)_2_, 1H); 1.61 (m, –C**H**_**2**_–CCOO, 2H); 1.51 (q, –CH–C**H**_2_–CH(CH_3_)_2_, not determined); 1.48 (m, –OCHC**H**_2_, not determined) (m 1.25 (m, –C**H**_**2**_–, 24H); 0.92 (d, CH–(C**H**_3_)_2_, 6H); 0.85 (m, –**CH**_**3**_, 3H).

## Author contributions

Conceptualization, R. C. S.; methodology, R. C. S.; formal analysis, Y. B. K.; investigation, Y. B. K.; resources, R. C. S.; data curation, Y. B. K.; writing – original draft preparation, Y. B. K.; writing – review and editing, all authors; supervision, A. D. S. and R. C. S.; funding acquisition, A. D. S. and R. C. S. All authors have read and agreed to the published version of the manuscript.

## Conflicts of interest

There are no conflicts to declare.

## Supplementary Material

RA-016-D6RA02359B-s001

## Data Availability

The data supporting this article have been included as part of the supplementary information (SI). Supplementary information: TGA curves and ^1^H NMR spectrum provided. See DOI: https://doi.org/10.1039/d6ra02359b.
