# Supplementary material for: Brown grease-derived ester base oils: tunable viscosity and cold-flow behaviour
Source: RSC Adv. 2026 Jul 3;16(35):36359–67. doi: 10.1039/d6ra02359b (PMC13330713; doi:10.1039/d6ra02359b)
Supplement: RA-016-D6RA02359B-s001 [file RA-016-D6RA02359B-s001.pdf]

Supplementary Information (SI) for RSC Advances.
This journal is © The Royal Society of Chemistry 2026

Supplementary Information

Brown Grease-Derived Ester Base Oils: Tunable Viscosity and Cold-Flow Behaviour

Ykok B. Ksor[a], Ashlyn D. Smith[a] and Rhett C. Smith[a*]

[a] *Department of Chemistry, Clemson University, Clemson, SC, 29634, USA.*

Fatty Acid:	Amount (mg per gram:)	% By Weight:
C4:0 - Methyl Butyrate	0.00	0.00
C6:0 - Methyl Caproate	0.10	0.01
C8:0 - Methyl Caprylate	0.75	0.08
C10:0 - Methyl Caprate	0.62	0.07
C12:0 - Methyl Laurate	1.02	0.11
C14:0 - Methyl Myristate	6.33	0.70
C14:1 - Methyl Myristoleate	1.33	0.15
C15:0 - Methyl Pentadecanoate	0.85	0.09
C16:0 - Methyl Palmitate	196.36	21.67
C16:1 - Methyl Palmitoleate	44.70	4.93
C18:0 - Methyl Stearate	56.96	6.29
C18:1 - Methyl Oleate	406.44	44.85
C18:2n6 - Methyl Linoleate	159.89	17.65
C18:3n6 - Methyl Gamma Linolenate	1.66	0.18
C18:3n3 - Methyl Linolenate	9.40	1.04
C18:4n3 - Methyl Stearidonate	0.90	0.10
C20:0 - Methyl Arachidate	2.04	0.23
C20:1 - Methyl Eicosenoate	4.41	0.49
C20:2n6 - Methyl 11-14 Eicosadienoate	1.82	0.20
C20:3n6 - Methyl Dihomo-gamma-Linolenoate	1.49	0.16
C20:4n6 - Methyl Arachidonate	3.99	0.44
C20:3n3 - Methyl Eicosatrienoate	0.10	0.01
C20:4n3 - Methyl Arachidonate	0.04	0.00
C20:5n3 - Methyl Eicosapentaenoate (EPA)	0.13	0.01
C22:0 - Methyl Behenate	1.05	0.12
C22:1 - Methyl Erucate	0.69	0.08
C22:2n6 - Methyl Docosadienoate	0.39	0.04
C21:5n3 - Methyl Heneicosapentaenoate	0.00	0.00
C22:4n6 - Methyl Docosatetranoate	0.89	0.10
C22:5n6 - Methyl Docosapentaenoate	0.22	0.02
C22:5n3 - Methyl Docosapentaenoate	0.34	0.04
C22:6n3 - Methyl Docosahexaenoate (DHA)	0.26	0.03
C24:0 - Methyl Lignocerate	0.59	0.06
C24:1 - Methyl Nervonate	0.37	0.04
**Total (mg/g)**	**906.11**	**100.00**

Fatty Acid Composition:	Amount (mg per gram:)	% By Weight:
Saturated	266.67	29.43
Monosaturated	457.93	50.54
Polyunsaturated	181.51	20.03
Trans Fatty Acids	0.00	0.00

Table 1. Fatty acid composition of brown grease determined by Lipid Analytical Laboratories using modified AOCS Official Method Ce 1i-07.

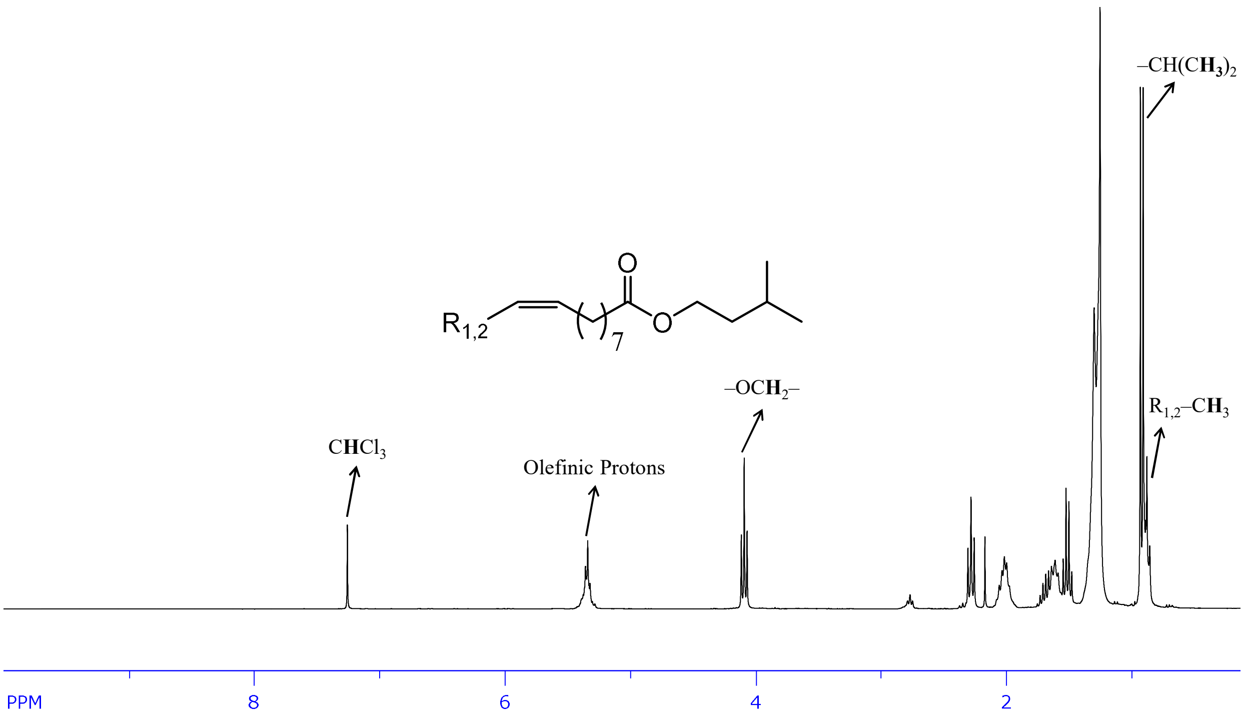

Figure S1. Proton NMR spectrum (300 MHz, $CDCl_3$) of **FAPEc**.

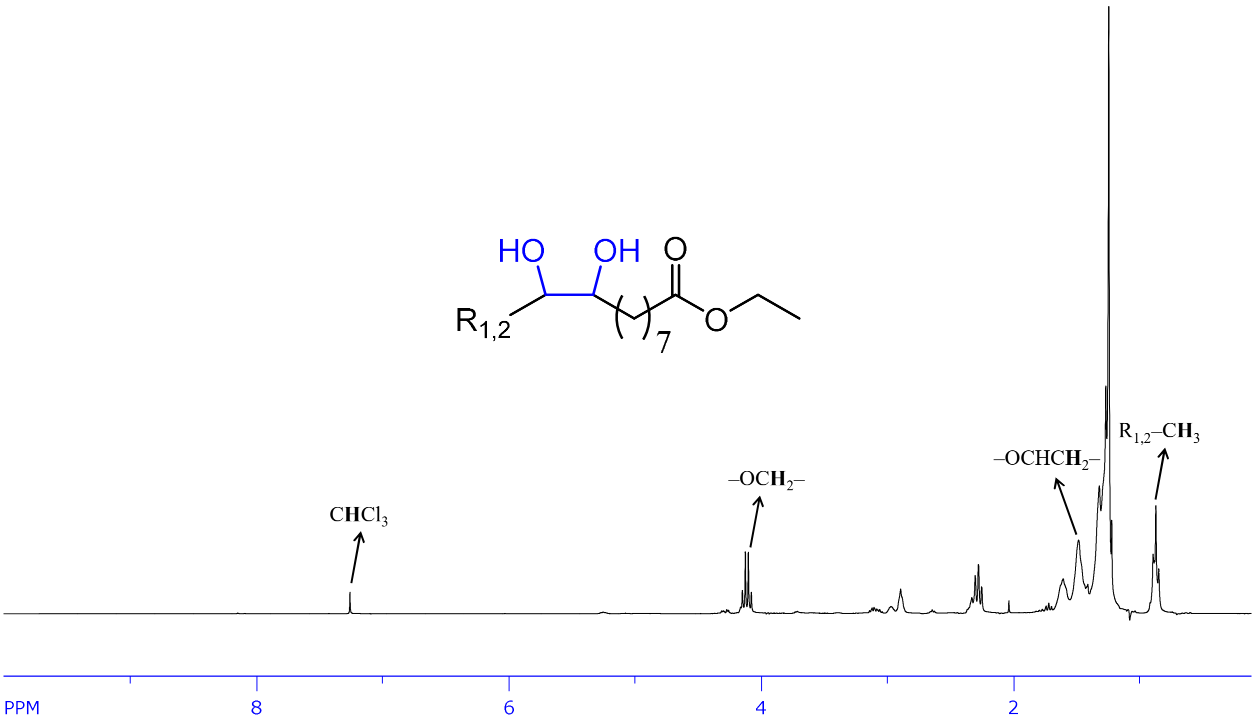

Figure S2. Proton NMR spectrum (300 MHz, $CDCl_3$) of **h-FAEEc**.

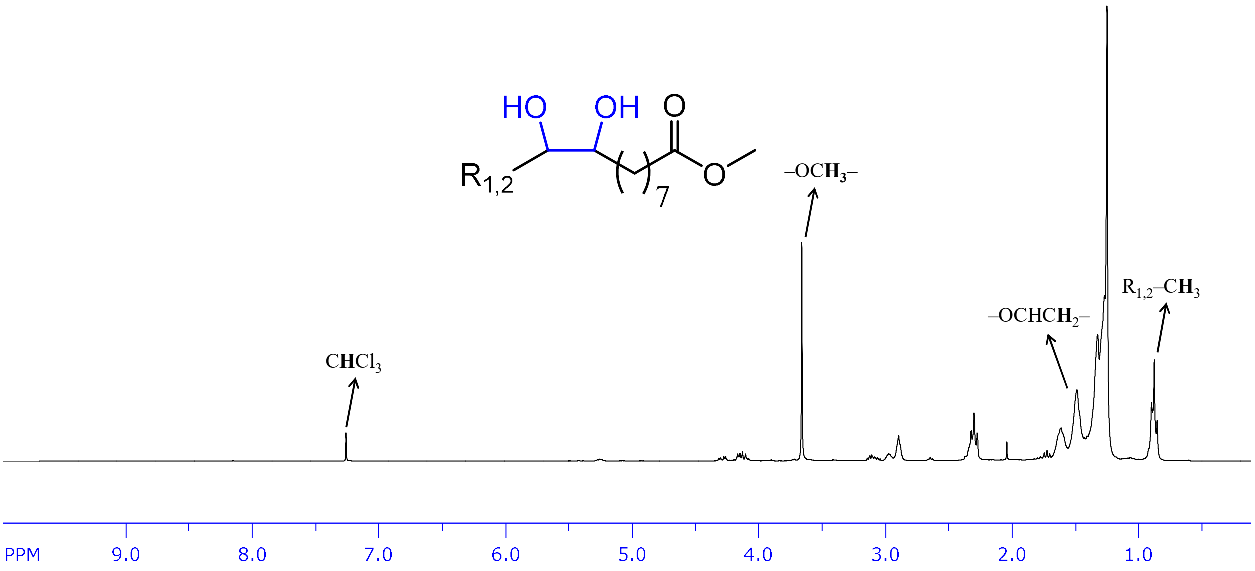

Figure S3. Proton NMR spectrum (300 MHz, $CDCl_3$) of **h-FAMEc**.

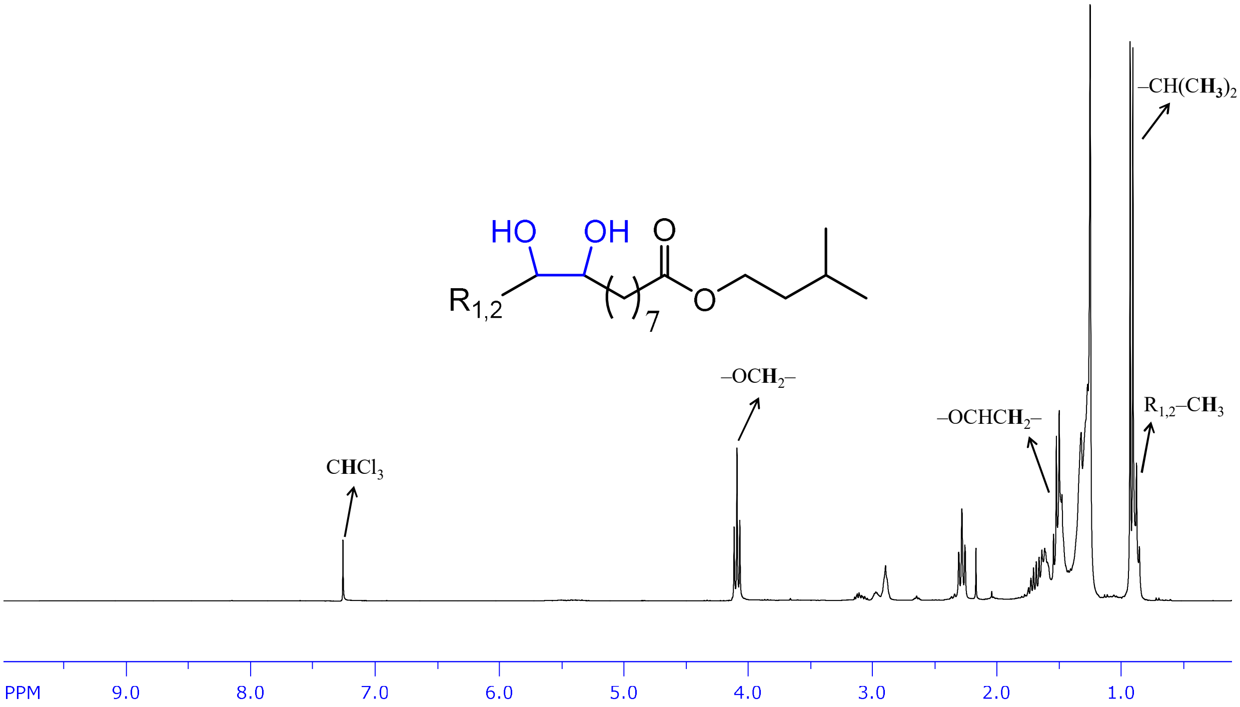

Figure S4. Proton NMR spectrum (300 MHz, $CDCl_3$) of **h-FAPEc**.

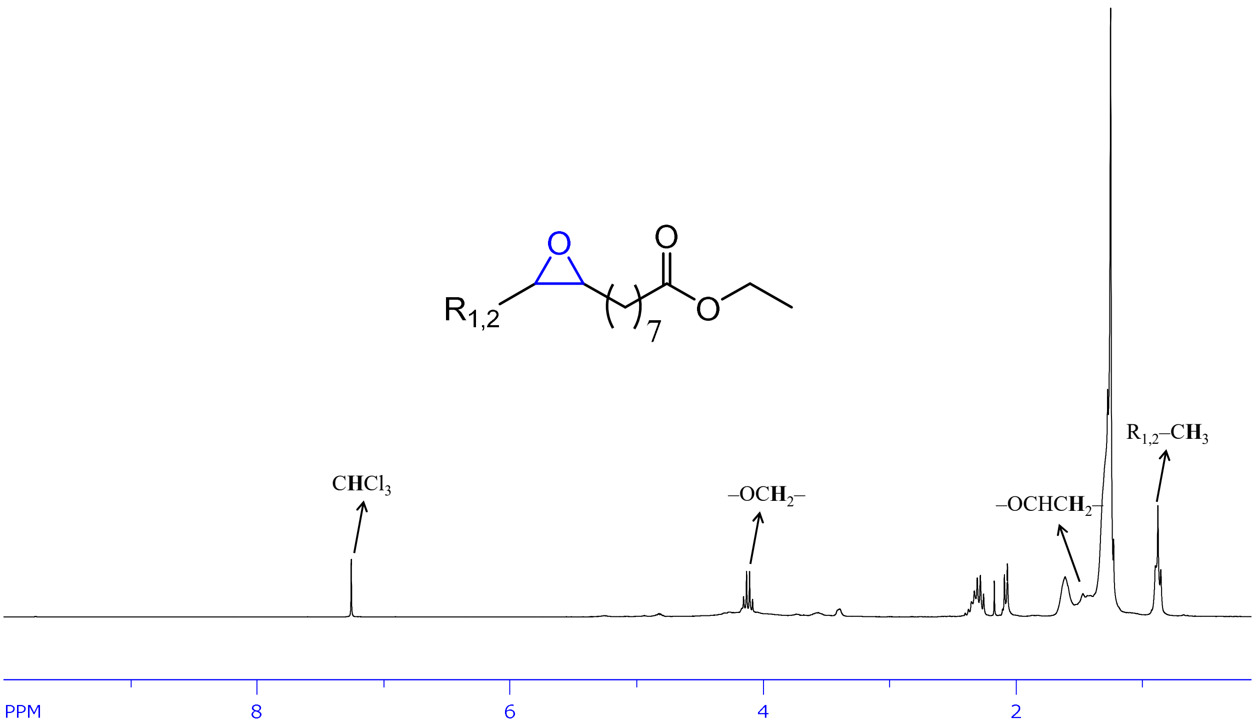

Figure S5. Proton NMR spectrum (300 MHz, $CDCl_3$) of **e-FAEEc**.

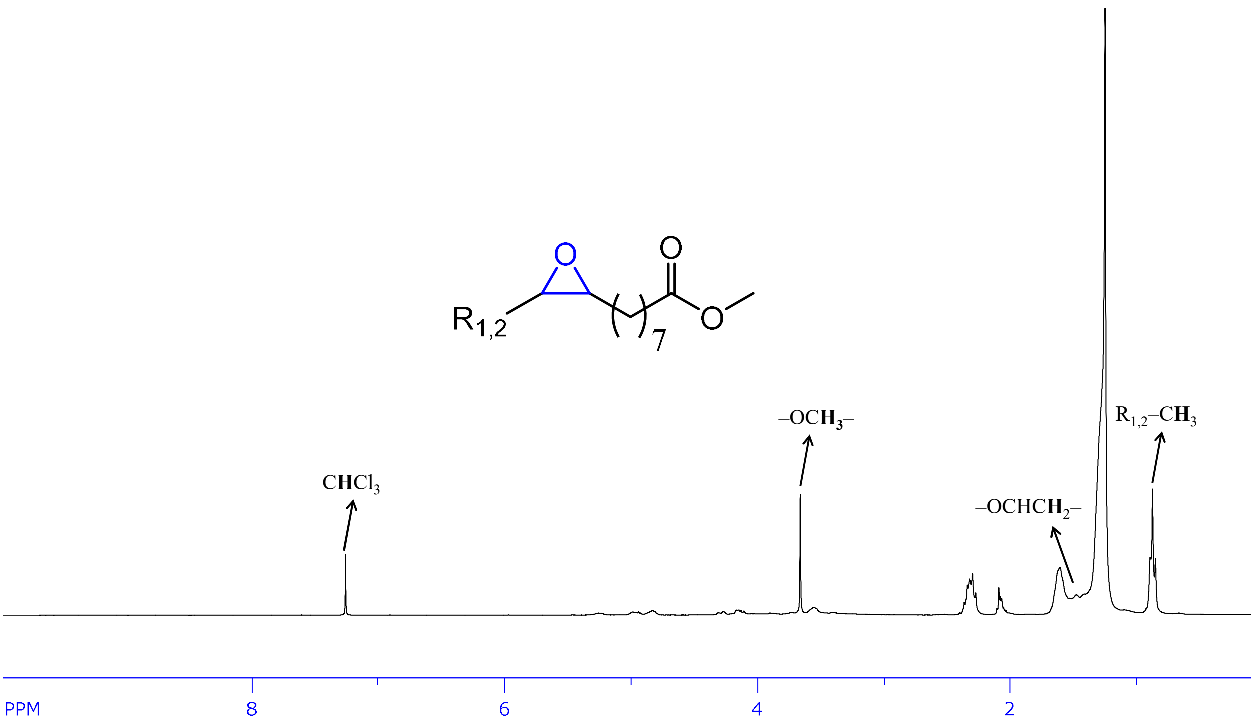

Figure S6. Proton NMR spectrum (300 MHz, $CDCl_3$) of **e-FAMEc**.

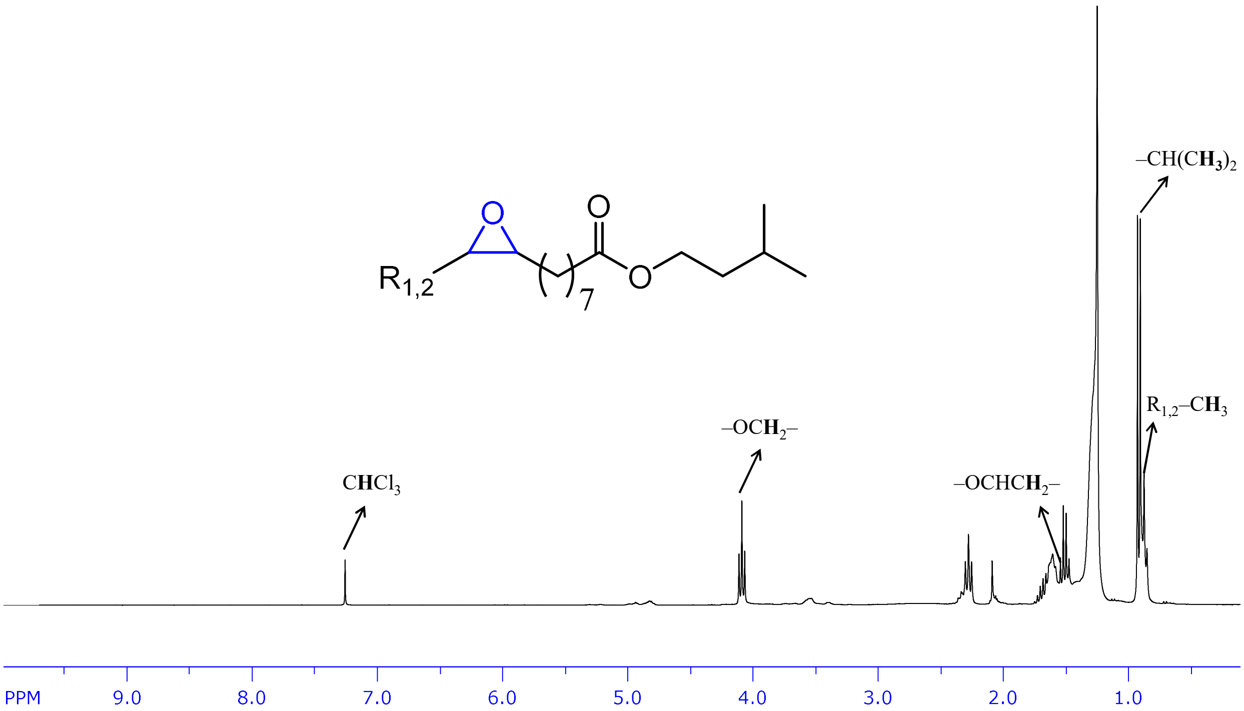

Figure S7. Proton NMR spectrum (300 MHz, $CDCl_3$) of **e-FAPEc**.

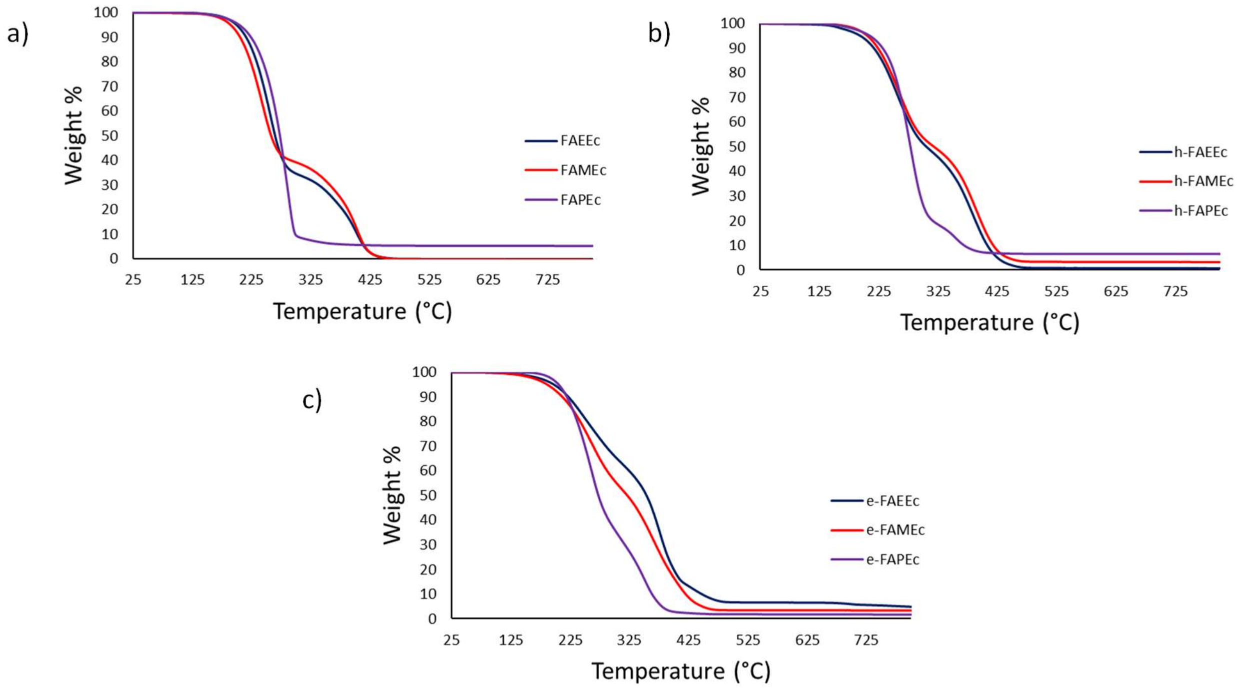

Figure S8. Mass loss curves from TGA for a) **FAEEc**, **FAMEc**, **FAPEc**, b) **h-FAEEc**, **h-FAMEc**, **h-FAPEc**, and c) **e-FAEEc**, **e-FAMEc**, **e-FAPEc**.

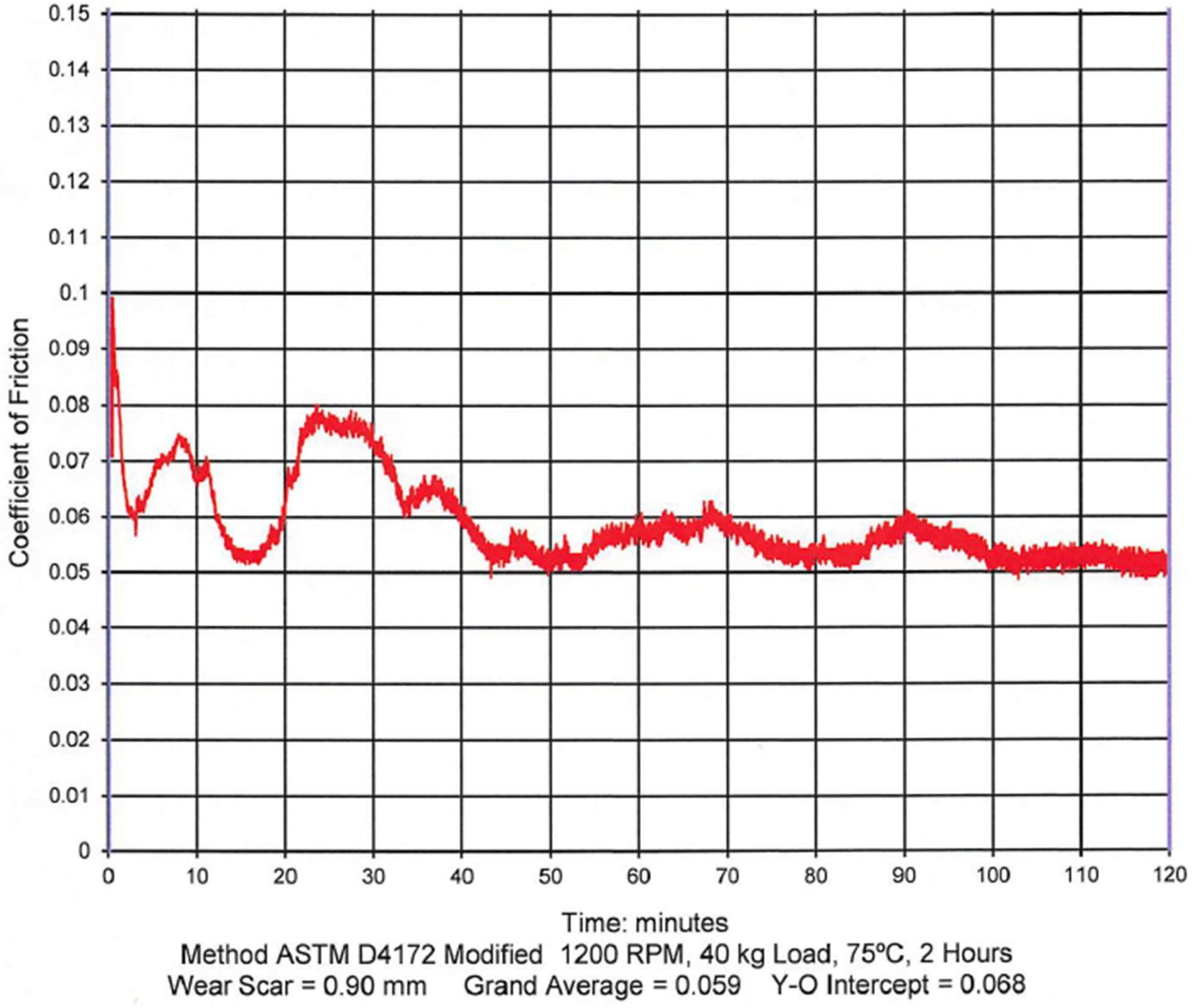

Figure S9. Coefficient of friction from four-ball wear testing of **h-FAEEc**. An average wear scar diameter of 0.90 mm was observed after this test.

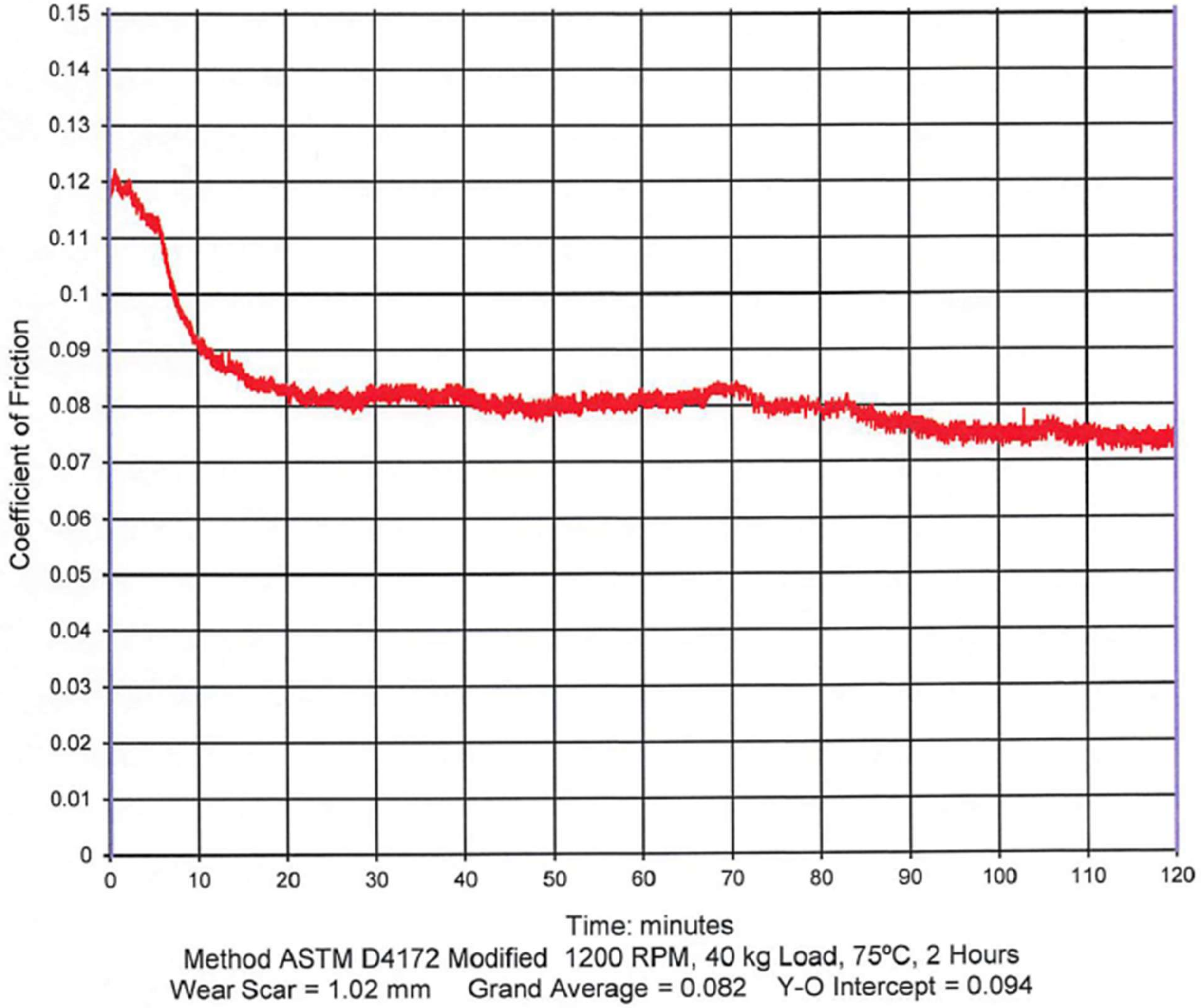

Figure S10. Coefficient of friction from four-ball wear testing of **FAPEc**. An average wear scar diameter of 1.02 mm was observed after this test.

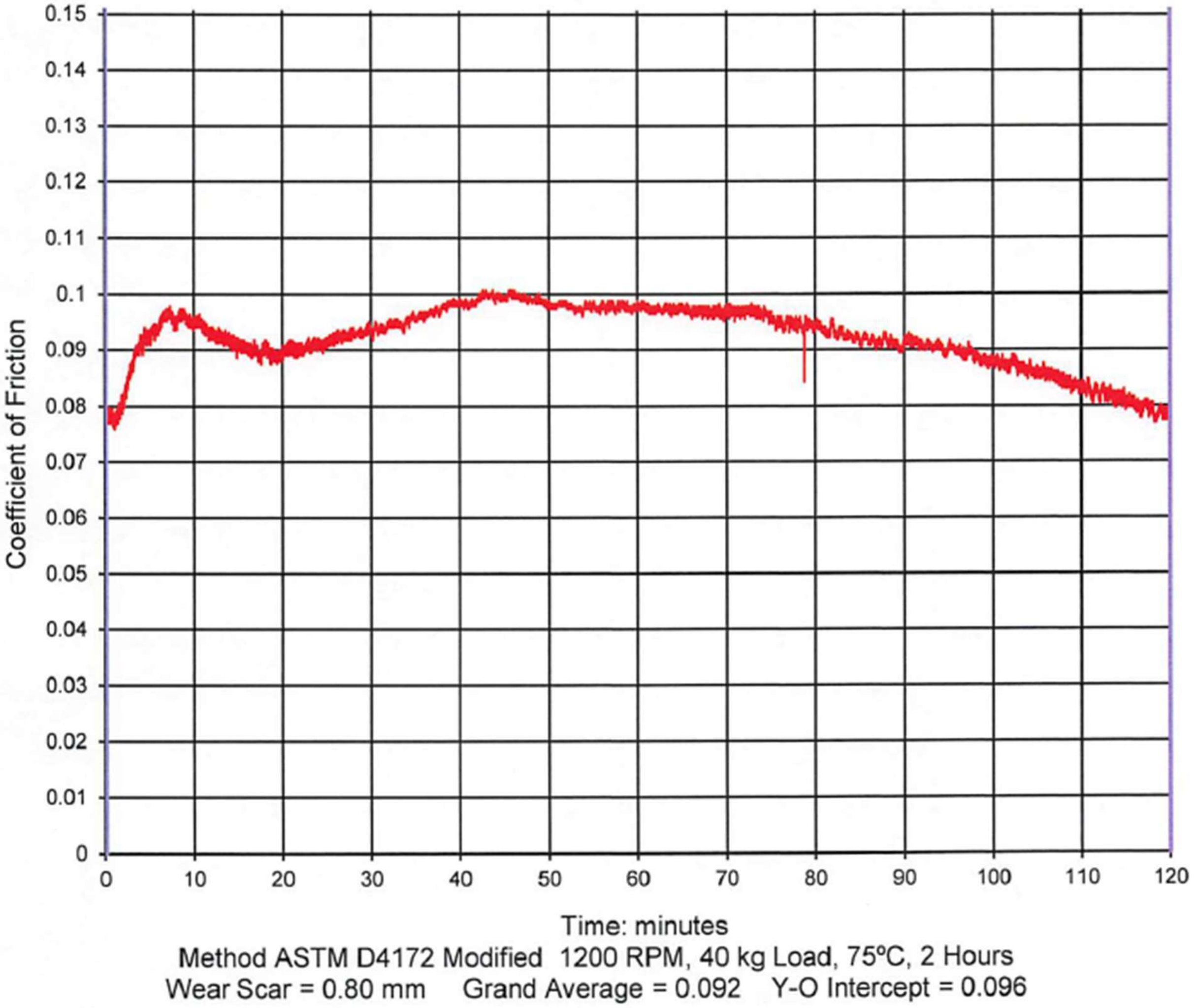

Figure S11. Coefficient of friction from four-ball wear testing of **e-FAPEc**. An average wear scar diameter of 0.80 mm was observed after this test.
